# Effect of Indocyanine Green-Guided Lymphadenectomy During Gastrectomy on Survival: Individual Patient Data Meta-Analysis

**DOI:** 10.3390/cancers17060980

**Published:** 2025-03-14

**Authors:** Matteo Calì, Alberto Aiolfi, Sho Sato, Jawon Hwang, Gianluca Bonitta, Francesca Albanesi, Giulia Bonavina, Marta Cavalli, Giampiero Campanelli, Antonio Biondi, Luigi Bonavina, Davide Bona

**Affiliations:** 1 I.R.C.C.S. Ospedale Galeazzi–Sant’Ambrogio, Division of General Surgery, Department of Biomedical Science for Health, University of Milan, 20122 Milan, Italy; matteocali94@gmail.com (M.C.); marta.cavalli@grupposandonato.it (M.C.); giampiero.campanelli@grupposandonato.it (G.C.); davide.bona@unimi.it (D.B.); 2 Department of Surgery, Minimally Invasive UGI Surgery and Oncology, Yokohama City University Gastroenterological Center, Yokohama 232-0024, Japan; shosato@yokohama-cu.ac.jp; 3 Minimally Invasive UGI Surgery, Severance Hospital–Division of General Surgery, Seoul 03722, Republic of Korea; june29@yuhs.ac; 4 Department of Oncologic Surgery 1–HPB, Division of General Surgery, Fondazione I.R.C.C.S Istituto Nazionale dei Tumori, 20133 Milan, Italy; francesca.albanesi@unimi.it; 5 Department of Obstetrics and Gynecology, IRCCS MultiMedica, 20138 Milan, Italy; giulia.bonavina@multimedica.it; 6 G. Rodolico Hospital, Surgical Division, Department of General Surgery and Medical Surgical Specialties, University of Catania, 95131 Catania, Italy; abiondi@unict.it; 7 IRCCS Policlinico San Donato, Division of General and Foregut Surgery, Department of Biomedical Sciences for Health, University of Milan, 20097 Milan, Italy; luigi.bonavina@unimi.it

**Keywords:** gastrectomy, lymphadenectomy, indocyanine green, overall survival, disease-free survival

## Abstract

Background: Indocyanine green-guided (ICG-guided) lymphadenectomy during gastrectomy for cancer has been proposed to enhance the accuracy of lymphadenectomy. The impact of ICG-guided lymphadenectomy on patient survival remains debated. Methods: The findings of the systematic review were reconstructed into an individual patient data (IDP) meta-analysis with restricted mean survival time difference (RMSTD). Overall survival (OS) and disease-free (DFS) survival were primary outcomes. RMSTD, standardized mead difference (SMD), and 95% confidence intervals (CI) were used as pooled effect size measures. Results: Three studies (6325 patients) were included; 42% of patients underwent ICG-guided lymphadenectomy. The patients’ age ranged from 47 to 72 years and 58% were males. Proximal, distal, and total gastrectomy were completed in 6.8%, 80.4%, and 12.8% of patients, respectively. The surgical approach was laparoscopic (62.3%) and robotic (37.7%). ICG-guided lymphadenectomy was associated with a higher number of harvested lymph nodes compared to non-ICG-guided lymphadenectomy (SMD 0.50; 95% CI 0.45–0.55). At the 42-month follow-up, OS and DFS estimates for ICG-guided vs. non-ICG-guided lymphadenectomy were 0.5 months (95% CI −0.01, 1.1) and 1.3 months (95% CI 0.39, 2.15), respectively. Conclusions: Our analysis suggests that ICG-guided lymphadenectomy offers equivalent long-term OS and DFS compared to non-ICG-guided lymphadenectomy.

## Simple Summary

Indocyanine green-guided (ICG-guided) lymphadenectomy during gastrectomy for cancer has been proposed to improve the accuracy of lymphadenectomy. The aim of this study is to compare the effect of ICG-guided vs. non-ICG-guided lymphadenectomy on long-term survival. Three studies (6325 patients) were included; 42% of patients underwent ICG-guided lymphadenectomy. This preliminary meta-analysis suggests that ICG-guided lymphadenectomy offers equivalent long-term OS and DFS compared to non-ICG-guided lymphadenectomy.

## 1. Introduction

Gastric cancer (GC) ranks as the fifth leading cause of cancer-related deaths globally [1,2]. In recent years, advancements in new therapies and a multimodal approach have transformed the management of GC. Nevertheless, surgical resection with adequate lymphadenectomy continues to be the cornerstone of treatment [3,4]. Lymph node (LN) involvement is a critical prognostic factor for survival. Factors influencing LN involvement include tumor location, submucosal infiltration, poor differentiation, large tumor size, and ulceration [5,6]. D2 lymphadenectomy is widely regarded as the standard for curative gastrectomy. While it has been successfully performed for decades in Eastern countries, D1 lymphadenectomy is more common in the West [7,8]. The theoretical benefits of D2 lymphadenectomy include a greater number of resected lymph nodes, which contribute to more accurate staging, the removal of potential metastatic nodes, and a lower risk of locoregional recurrence [9,10,11]. The eighth edition guidelines from the International Union for Cancer Control/American Joint Committee on Cancer state that retrieving more than 15 lymph nodes is necessary for reliable staging [12]. Additionally, studies indicate that removing over 30 lymph nodes correlates with improved long-term survival [13]. Thus, determining the optimal extent of lymph node dissection in gastric cancer patients, with goals of preventing understaging or undertreatment, reducing complications, and enhancing survival, remains an area of active discussion.

Indocyanine green (ICG) has emerged as valuable tracer for mapping lymphatic pathways during lymphadenectomy in gastric cancer surgeries. Its fluorescence properties enable real-time visualization of lymphatic vessels, thus improving the surgeon’s ability to accurately excise LNs while safeguarding healthy tissue. The application of ICG in gastrectomy has been proposed in an attempt to enhance the accuracy of lymphadenectomy, increase the number of harvested LNs, reduce postoperative complications, and reduce the risk of recurrent lymph node metastasis. By enabling a more targeted approach, ICG might contribute to better outcomes in gastric cancer surgeries, representing a significant advancement in surgical oncology techniques [14,15,16,17]. However, the lack of high-quality evidence prevents definitive conclusions about its clinical significance [18]. Specifically, the long-term oncological effectiveness of ICG-guided lymphadenectomy in gastric cancer remains a topic of debate [19,20,21,22].

Hence, aim of this study is to evaluate the impact of ICG-guided compared to standard non-ICG-guided lymphadenectomy during gastrectomy on long-term patient survival.

## 2. Materials and Methods

A systematic review was conducted in accordance with the Preferred Reporting Items for Systematic Reviews and Meta-Analyses (PRISMA 2020) guideline (File S2) [23]. No ethical approval was necessary. Databases utilized included Scopus, MEDLINE, Web of Science, ClinicalTrials.gov, Cochrane Central Library, and Google Scholar [24]. The initial search was performed during March 2024 and updated on 1 November 2024. A combination of the following medical subject headings (MeSHs) terms was used: “Gastric Cancer”, “Gastric Carcinoma”, “Gastrectomy”, “Gastric resection”, “ICG”, “indocyanine”, “fluorescence”, “overall survival”, “disease free survival”, and “relapse free survival”. The comprehensive literature search strategy is illustrated in File S1. All titles were reviewed, appropriate abstracts were collected, and the reference lists of each article were evaluated independently by three authors (A.A., M.C., and F.A.). The study was registered with PROSPERO (CRD42024623233).

### 2.1. Eligibility Criteria

Inclusion criteria comprised (1) observational and randomized controlled trial (RCT) providing long-term survival data or Kaplan–Meier survival curves that compare ICG-guided vs. non-ICG-guided lymphadenectomy in the context of curative gastrectomy; (2) if multiple articles from the same institution, study group, or dataset were identified, preference was given to those with the longest follow-up duration or the largest sample size; (3) for duplicate studies, the most recent and comprehensive reports were chosen. Exclusion criteria included (1) studies not published in English; (2) studies that do not include a comparative analysis between ICG-guided and non-ICG-guided lymphadenectomy; (3) studies that failed to report the predefined primary outcomes; (4) studies that focused solely on short-term outcomes related to ICG-guided lymphadenectomy; (5) studies with fewer than 20 patients in each study group.

### 2.2. Data Extraction

The following data points were collected: authors, publication year, country, study design, number of patients, gender, age (years), body mass index (BMI), surgical procedure, tumor characteristics, tumor location, total number of harvested and metastatic LNs, neoadjuvant or adjuvant therapy, pathological outcomes, duration of follow-up, and long-term patients’ survival. Additional information included the dose of ICG, injection methodology, timing and detection software. Two authors (A.A. and M.C.) independently gathered all data and reconciled any discrepancies during the evaluation. A third author (D.B.) subsequently reviewed the database to address any inconsistencies.

### 2.3. Outcomes of Interest and Definition

The primary outcomes were overall survival (OS) and disease-free survival (DFS). Secondary outcomes were the total number of harvested LNs and the total number of metastatic LNs. OS was defined as the time from surgery to the last known follow-up or death, while DFS was defined as the period from surgical resection to the onset of local recurrence or death. Data on OS and DFS were obtained from Kaplan–Meier survival curves.

### 2.4. Quality Assessment and Assessment of Certainty of Evidence

Two authors (M.C. and A.A.) independently evaluated the methodological quality of the included studies using the ROBINS-I and ROB2 tools [25,26]. They considered factors such as confounding, selection, classification, intervention, missing data, outcome measurement, and reporting bias, categorizing each domain as “low”, “moderate”, “serious”, or “critical”. The classification of confounding bias for each study was organized into these risk categories [27]. The quality of evidence across studies was evaluated using the Grading of Recommendations, Assessment, Development, and Evaluation (GRADE) tool [28]. GRADE evidence profiles for each comparison and outcome were generated with GRADEpro software version GDT (https://www.gradepro.org; accessed on 30 November 2024). The certainty of the evidence was determined by factors such as the risk of bias across studies, incoherence, indirectness, imprecision, publication bias, and other relevant considerations [29].

### 2.5. Statistical Analysis

The findings from the systematic review were qualitatively summarized and converted into a frequentist meta-analysis of the restricted mean survival time difference (RMSTD) [30,31]. Individual patient time-to-event data (IPD) were reconstructed from Kaplan–Meier curves [32] using the Get Data Graph Digitizer software version 5.0 (https://automeris.io; accessed on 1 December 2024). The pooled RMSTD was calculated using a random-effects multivariate meta-analysis, which utilized strength across time points while accounting for within-trial covariance. Additionally, a flexible hazard-based regression model was created using IPD, which included a normally distributed random intercept. To model the baseline hazard within the periocular, an exponential of a cubic B-spline (degree 3) without interior knots was used, with model selection based on the Akaike information criterion (AIC). Time-varying effects of surgical treatment were represented through interaction terms between the surgical intervention and the baseline hazard, evaluated with likelihood ratio tests. The hazard function plot was generated using marginal predictions [33].

The frequentist random effect methodology was used to assess the other oncological outcomes through the standardized mean difference (SMD) estimation [34,35]. An inverse-variance method and DerSimonian–Laird estimator for the variance of the true effect size (τ^2^) were performed [36]. Heterogeneity among studies was evaluated by the I^2^ index and Cochran’s Q test [37]. Statistical heterogeneity was considered low, moderate, and high for I^2^ values of 25, 50, and 75%, respectively [37,38]. Statistical significance was defined by two-sided *p*-values below 0.05, and confidence intervals were computed at 95%. The statistical analysis was conducted using the R software application (version 3.2.2; R Foundation, Vienna, Austria) [39].

## 3. Results

### 3.1. Systematic Review

**Figure 1:**
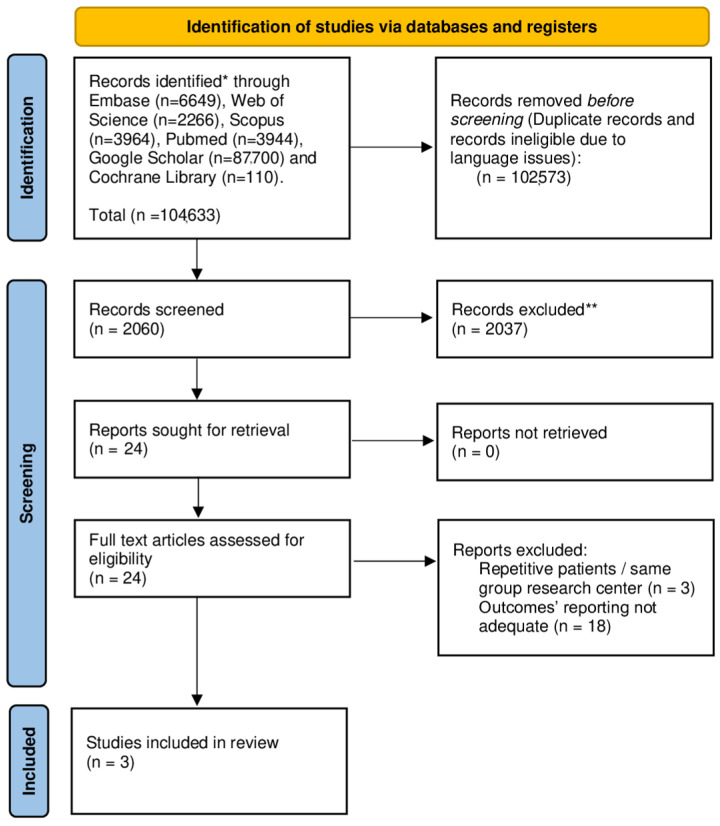
The Preferred Reporting Items for Systematic Reviews and Meta-Analyses (PRISMA) diagram. * ”Gastric Cancer”, “Gastric Carcinoma”, “Gastrectomy”, “Gastric resection”, “ICG”, “indocyanine”, “fluorescence”, “ overall survival”, “disease free survival”, “relapse free survival”. ** Records excluded by title and abstract screening due to publication type (publications such as conference abstracts, book chapters, and conference posters were excluded) and unrelated topics.

The selection process flowchart is presented in Figure 1. Initially, 2060 publications were screened after eliminating duplicates, leading to 24 papers selected for full-text review. Following evaluation and the removal of studies with partial overlap, three papers fulfilled the inclusion and exclusion criteria and were included in the quantitative analysis. Of these, two were observational studies, and one was an RCT. The quality assessment of the included studies is illustrated in Supplementary Figure S1A,B.

A total of 6325 patients who underwent gastrectomy for cancer were included for quantitative analysis (Table 1). ICG-guided lymphadenectomy was performed in 42% of patients (n = 2633). The patients’ ages ranged from 47 to 72 years, with 57.9% being males. Preoperative BMI varied from 20.5 to 26.7 kg/m². The pathological tumor staging system varied among the studies. Tumor location was specified in two studies and was distributed in the gastric fundus (14.7%), gastric body (30.5%), and antrum (54.8%). All studies described a four-quadrant preoperative endoscopic ICG injection. The timing and dosage differed across the studies, while details regarding the ICG brand and intraoperative detection software are provided in Supplementary Table S1. Proximal, distal, and total gastrectomy were completed in 6.8%, 80.4%, and 12.8% of patients, respectively. The surgical approach was laparoscopic (62.3%) and robotic (37.7%). All studies specified the extent of lymphadenectomy; two studies involved D2 lymphadenectomy [40,41], while one mentioned both D1+ and D2 lymphadenectomy [42]. The total number of harvested LNs was specified in three studies while two studies reported the number of metastatic nodes over the total. Postoperative complications, stratified according to the Clavien–Dindo classification, were specified in two studies [40,42]. None of the studies reported on neoadjuvant treatment, as this was a predefined exclusion criterion. Adjuvant treatment was reported in one study [41] and completed in 55.8% of patients. None of the studies specified HER-2, PD-L1, or microsatellite status.

### 3.2. Meta-Analysis—Primary Outcomes

**Figure 2:**
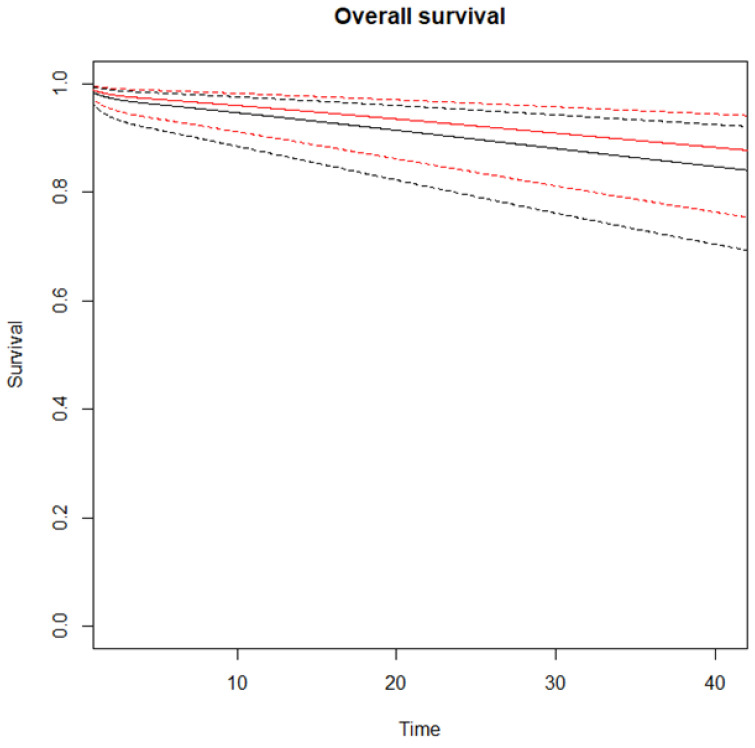
Estimated pooled OS (Y-axis) for ICG-guided (red line) versus no-ICG guided lymphadenectomy (black line). Time (X-axis) is expressed in months. Continuous lines indicate survival curves with 95% confidence intervals (dotted lines).

The RMSTD OS clinical appraisal was estimated in all three studies [40,41,42] for the comparison between ICG-guided vs. non-ICG-guided lymphadenectomy (Supplementary Figure S2). The RMSTD time-line estimation at different time points is reported in Table 2. At 42 months, the combined effect is 0.5 months (95% CI −0.01, 1.1), indicating that patients that underwent ICG-guided lymphadenectomy lived 0.5 months more on average compared to non-ICG-guided lymphadenectomy patients. The estimated pooled OS for ICG-guided and non-ICG-guided lymphadenectomy is depicted in Figure 2. Considering the non-proportional hazard model (*p* < 0.001), the time-varying hazard ratios for ICG-guided vs. non-ICG-guided lymphadenectomy are depicted in Supplementary Figure S3.

**Figure 3:**
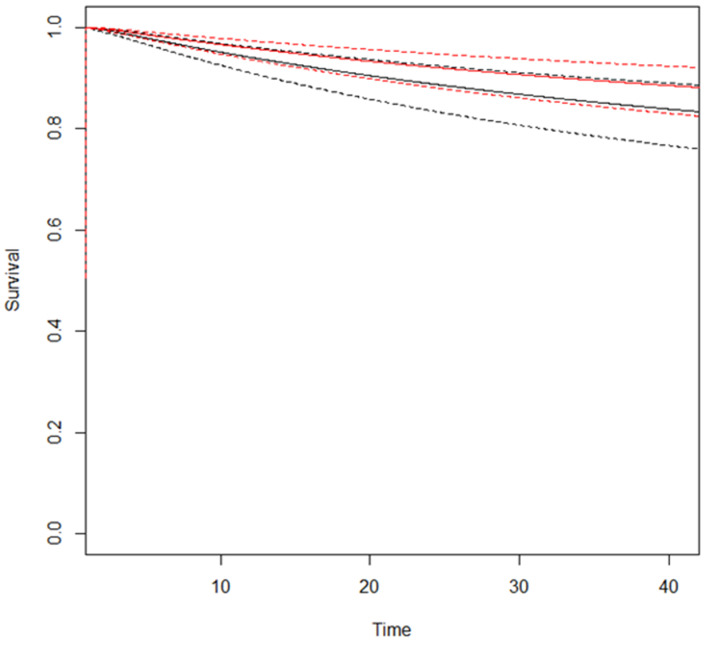
Estimated pooled DFS (Y-axis) for ICG-guided (red line) versus no-ICG guided lymphadenectomy (black line). Time (X-axis) is expressed in months. Continuous lines indicate survival curves with 95% confidence intervals (dotted lines).

The RMSTD DFS clinical appraisal was estimated from all three studies (Supplementary Figure S4) for the comparison between ICG-guided vs. non-ICG-guided lymphadenectomy at different time points (Table 3). At 42 months, the combined effect is 1.3 months (95% CI 0.39, 2.15), indicating that at 42 months patients that underwent ICG-guided lived 1.3 months more on average compared to non-ICG-guided lymphadenectomy patients. The estimated pooled DFS for ICG-guided and non-ICG-guided lymphadenectomy is represented in Figure 3. Considering the non-proportional hazard model (*p* < 0.001), the time-varying hazard ratios for ICG-guided vs. non-ICG-guided lymphadenectomy are depicted in Supplementary Figure S5.

### 3.3. Secondary Outcomes

The total number of harvested LNs was reported in all included studies (6325 patients). The quantitative analysis shows significantly higher total number of harvested LNs for ICG-guided vs. non-ICG-guided lymphadenectomy (SMD 0.50; 95% CI 0.45, 0.55; I^2^ = 0.0%). The sensitivity analysis confirms the robustness of these findings in terms of point estimation, 95% confidence intervals, and heterogeneity. The number of metastatic LNs was reported in two studies [40,41]; therefore, a quantitative assessment was not feasible.

### 3.4. Certainty of Evidence

A complete evaluation of the certainty of evidence and the considerations for grading are provided in Supplementary Table S2. Moderate certainty of evidence was observed for OS and DFS.

## 4. Discussion

This individual patient data meta-analysis suggests that ICG-guided has equivalent OS and DFS compared to non-ICG-guided lymphadenectomy with follow-up data extending up to 42 months. Given the limited number of studies included and the potential for selection bias, caution is essential to prevent misinterpretation of the findings. Thus, our results should be regarded as preliminary and plea for further confirmation through more extensive and rigorous research.

GC ranks as the sixth most common cancer and the third leading cause of cancer mortality globally [1,2]. Key risk factors include genetic predisposition, alcohol consumption, smoking, and Helicobacter pylori infection. In cases of resectable GC, a multimodal treatment combining surgery and systemic therapy has been shown to be associated with improved long-term disease-specific survival. Surgical resection with lymphadenectomy represents the mainstay for curative treatment [3,4,43,44]. The debate over lymph node dissection extent, comparing extended (D2 or D3) versus limited (D1) dissection, has persisted. Eastern surgeons favor more extended dissections to mitigate the risk of lymphatic dissemination, theorizing that this could enhance survival by refining disease staging and eliminating microscopic metastases [45,46,47]. Notably, earlier studies indicated potential survival benefits for D2 lymphadenectomy [11,48,49,50]. A 2015 Cochrane review found no significant differences in 5-year OS or DFS for D2 vs. D1, but observed significantly better disease-specific survival for D2 [51]. A 2021 meta-analysis suggested trends toward improved 5-year OS in patients classified as T3 and N+ who underwent D2 [52]. Consequently, the NCCN and ESMO guidelines endorse D2 lymphadenectomy as the optimal curative intervention for potentially operable gastric cancer with at least 16 regional LNs for pathological examination [3,5]. However, the routine application of this extended dissection correlates with higher postoperative complications and mortality, without consistent evidence of improved survival in RCTs and meta-analysis [47,53,54,55,56,57,58].

In response to the need for precision and reproducibility in lymphadenectomy, ICG has been introduced as a reliable tool for lymphatic mapping. ICG is a water-soluble tricarbocyanine dye with an excitation spectrum between 700 and 850 nm, peaking at emission wavelengths of 810–830 nm when exposed to near-infrared light [59]. Upon injection, ICG distributes according to plasma volume, demonstrating minimal tissue binding and a high affinity for plasma proteins. This characteristic facilitates its transport to the lymphatic system, effectively highlighting LNs. Initially utilized for detecting sentinel LNs in early gastric cancer [60,61], the application of ICG has expanded to marking tumor locations, ensuring disease-free resection margins [62], and guiding lymphadenectomy during gastrectomy [16,17,18]. ICG lymphatic mapping might serve as a useful tool for identifying lymph glands and lymphatics, enabling more precise lymphadenectomy and potentially reducing non-compliance rates. However, despite its advantages, there is currently no standardized protocol regarding dosage, injection methodology, or timing, which complicates consistency in clinical practice. Additionally, a significant limitation of ICG fluorescence is that it primarily indicates lymphatic drainage patterns related to tumors rather than directly identifying metastatic LNs. This limitation arises from the potential obstruction of lymphatic vessels by cancer cells, disrupting the normal flow of ICG and obscuring the presence of metastatic involvement in the LNs. Jung et al. [15] quantified this limitation, noting an accuracy range of 62% to 97% and a false-negative rate of 46% to 60% in patients who did not undergo neoadjuvant treatments. Notably, it has been stated that the accuracy and false-negative rates may significantly be different in patients who received prior neoadjuvant treatment [15].

ICG-guided lymphadenectomy has emerged as a valuable technology for increasing the number of harvested LNs during gastrectomy. A 2024 retrospective study by Kim et al. [42] reported that ICG-guided lymphadenectomy significantly increased the number of retrieved LNs (48 vs. 39; *p* < 0.001), with higher counts both in the perigastric (30 vs. 24; *p* < 0.001) and extraperigastric (15.5 vs. 13; *p* < 0.001) stations. The study also noted a greater proportion of patients achieving 16 or more harvested LNs (99.5% vs. 98.1%; *p* < 0.001) and an increased number of patients with 30 or more LNs (86.6% vs. 72.2%; *p* < 0.001). Similarly, research by Park et al. [63] concluded that ICG-guided lymphadenectomy led to a significantly higher total number of retrieved nodes (56 vs. 46; *p* < 0.001) compared to non-ICG-guided lymphadenectomy. Moreover, Kwon and colleagues [16] found an increase in the overall number of retrieved LNs (48.9 vs. 35.2; *p* < 0.001), particularly from perigastric stations (No. 2, 6, 7, 8, and 9). These findings align with our results, which confirmed a significantly higher number of harvested LNs with ICG-guided lymphadenectomy (SMD 0.50; 95% CI 0.45, 0.55). The removal of a higher number of lymph nodes has been proposed to be associated with better tumor staging, improved locoregional control, reduced local recurrences, and, conceivably, improved survival [13].

However, the impact of ICG-guided lymphadenectomy on long-term survival is still debated. Our findings indicate that ICG-guided lymphadenectomy does not appear to impact long-term OS in patients with GC. Specifically, the 42-month RMSTD estimation was 0.5 months (95% CI −0.01, 1.1), indicating that patients that underwent ICG-guided lymphadenectomy tended to live 0.5 months more on average compared to non-ICG-guided lymphadenectomy patients. This aligns with Kim et al. [42], who reported similar 60-month OS (*p* = 0.189) in the two patient groups with comparable pathologic T and TNM stages. Similarly, Wei et al. [40] in their observational study concluded comparable medium-term OS (*p* > 0.05) for ICG-guided vs. non-ICG-guided lymphadenectomy. In contrast, the 2023 FUGES12 trial [41] reported a better OS in the ICG-guided arm compared to the non-ICG-guided lymphadenectomy patients both in the overall (86% vs. 73.6%; *p* = 0.015) and in the intention-to-treat (*p* = 0.014) analyses. In our study, the RMSTD DFS estimation supported an improved DFS in ICG-guided lymphadenectomy; specifically, at 42-month follow-up ICG-guided lymphadenectomy patients tended to live 1.3 months more on average compared to non-ICG patients. These results are consistent with the FUGES12 trial [41], which demonstrated significantly improved DFS in patients undergoing ICG-guided lymphadenectomy (81.4% vs. 68.2%; *p* = 0.012). Importantly, the authors reported a significant difference in the cumulative incidence of locoregional recurrence (1.6% vs. 7.8%; *p* = 0.048); however, no differences were observed in the cumulative incidence of recurrence in the peritoneum, liver, or multiple sites. In contrast, Kim et al. [42] failed to conclude a significant impact of ICG-guided lymphadenectomy on DFS (*p* = 0.161). Our findings indicate that although a greater number of LNs were harvested during surgery, the real-world clinical impact on long-term OS and DFS appear limited. This could be attributed to the influence of various factors beyond just the number of harvested LNs on patient survival. These include the number of metastatic LNs, the specific lymph node stations involved and their prognostic significance (i.e., stations no. 4d and 6) [64,65,66,67], patient comorbidities, nutritional status at diagnosis, tumor stage, grading, histologic variants, postoperative complications, lymphatic invasion, genetic predisposition, molecular expression (i.e., HER2 and PD-L1), mismatch repair/microsatellite instability (MMR/MSI), claudins, and the response to perioperative treatments [68,69,70,71,72]. Further, although the topic is controversial, evidence suggests that the specialized expertise and experience of the operating surgeon can influence short-term and long-term survival outcomes in gastrectomy [73]. A study from South Korea revealed that surgeons who performed more than 100 cancer resections contributed to improved 5-year survival rates [74]. Similarly, Asplund et al. in their nationwide population-based cohort study found that conducting over 20 gastrectomies/year may positively affect 3–5-year postoperative mortality, significantly lowering the long-term mortality rate (12.4% vs. 8.6%). Furthermore, centralizing procedures at high-volume referral centers and increasing annual hospital volume could enhance 5-year survival rates and reduce recurrence rates [75]. Unfortunately, in the current analysis, information regarding the surgeons’ proficiency and the total number of gastric resections performed annually was not provided.

To our knowledge, this is the first IPD meta-analysis that evaluates the impact of ICG-guided lymphadenectomy technique on survival using the RMSTD methodology. This approach provides a more precise and effective time-dependent survival estimate than hazard ratios and odds ratios. The RMSTD methodology allows for a thorough assessment of the long-term survival effects of ICG-guided lymphadenectomy versus non-ICG-guided lymphadenectomy during follow-up. RMSTD is becoming increasingly acknowledged in clinical oncology for its reliability and clarity in measuring survival benefits. Compared to OR and HR, RMSTD offers a clearer interpretation of survival time and enables the analysis of more detailed individual-level data, resulting in more accurate survival estimates that enhance global comparisons and inform clinical decision-making [76]. Our data should be interpreted with caution due to several related limitations. First, the studies included were few and conducted in Eastern tertiary centers, which may limit the generalizability of the results to Western populations. Second, none of the patients in the included studies received neoadjuvant therapy; only one study reported on adjuvant treatments [32], and one study [41] excluded patients with preoperative evidence of bulky regional LNs (>3 cm). Third, there is heterogeneity in patient baseline characteristics, pathological stage distribution with low number of stage III patients, tumor location and types of surgical resection (proximal, distal, total), and technique for ICG injection, timing, and dosage. Fourth, only one of the three studies was an RCT [41], which mitigates potential selection bias. The other two studies were retrospective in design [40,42], which may introduce selection and publication bias despite the use of propensity score matching. Lastly, factors such as the variability in multidisciplinary perioperative care teams, different surgical approach (laparoscopy and robotic), differences in surgical techniques among operators, occurrence of postoperative complications [77], and genetic expression and molecular profiles must be considered due to their potential influence on long-term survival.

## 5. Conclusions

Our data indicates that while ICG-guided lymphadenectomy is associated with a significantly higher number of harvested LNs, its real-world clinical impact on long-term survival remains uncertain. Due to the limited number of studies included and the potential for selection or reporting bias, it is crucial to exercise caution to avoid misinterpretation. Therefore, our results should be considered preliminary and highlight the need for future rigorous, well-designed trials focusing on long-term survival.

## Figures and Tables

**Table 1:** Demographic and clinical characteristics of patients undergoing radical gastrectomy with ICG-guided (ICG) and non-ICG-guided lymphadenectomy. AJCC American Joint Committee on Cancer; years (yrs); body mass index (BMI); male (M); distal gastrectomy (DG); total gastrectomy (TG); proximal gastrectomy (PG); lymph nodes (LNs); retrospective (Ret); randomized controlled trial (RCT); inverse–probability-of-treatment weighting (IPTW); gastric fundus (F); gastric body (B); antrum (A); esophagogastric junction (EGJ); not reported (nr). Data are reported as numbers, mean ± standard deviation, median (range).

Author, Country, Year	Study Period	Study Design	Group	No. Pts	Age (yrs)	Gender (M)	BMI (kg/m^2^)	Surgical Procedure (DG/TG/PG)	Staging System	Tumor Location	Extent of Lymphadenectomy	Metastatic LNs	Total No. LNs
Wei et al., China, 2022 [40]	January 2018–August 2019	Ret	ICG	107	59.3 ± 8.9	57/50	24.6 ± 3.4	59/48/0	AJCC 8th	nr	D2	6.4 ± 10.9	49.5 ± 12.7
			no-ICG	88	61.5 ± 10.3	48/40	24.9 ± 2.6	41/47/0				3.3 ± 6.4	44.4 ± 10.2
Chen et al., China, 2023 [41]	November 2018–July 2019	RCT	ICG	129	57.8 (10.7)	86/43	23.2 (3.2)	nr	AJCC 7th	33F/21B/75A	D2	5.6 (11.2)	50.5 (15.9)
			no-ICG	129	60.1 (9.1)	87/42	22.8 (3.1)			66F/14B/49A		5.7 (8.9)	42.0 (10.3)
Kim et al., South Korea, 2024 [42]	January 2013–December 2021	Ret IPTW	ICG	2397	59.8 (12.3)	1409/988	23.6 (3.1)	1948/276/172	nr	303F/757B/1316A/27EGJ	1650 D1+ 747 D2	nr	48.4 (18.5)
			no-ICG	3475	59.5 (12.3)	1979/1496	23.6 (3.1)	2828/407/239		434F/1080B/ 1924A/37EGJ	2350 D1+ 1125 D2	nr	39.8 (16.3)

**Table 2:** Overall survival. The restricted mean survival time difference (RSMTD) estimation at different time horizons for ICG-guided vs. non-ICG-guided lymphadenectomy comparison. 95% CI: 95% confidence intervals; mos: months.

Time Horizon	No. Studies	RMSTD (mos)	95% CI	*p* Value
12 months	3	0.1	−0.20, 0.39	0.51
24 months	3	0.2	−0.14, 0.63	0.21
36 months	3	0.4	−0.01, 0.87	0.06
42 months	3	0.5	−0.01, 1.1	0.05

**Table 3:** Disease-free survival. The restricted mean survival time difference (RSMTD) estimation at different time horizons for ICG-guided vs. non-ICG-guided lymphadenectomy comparison. 95% CI: 95% confidence intervals; mos: months.

Time Horizon	No. Studies	RMSTD (mos)	95% CI	*p* Value
12 months	3	0.1	0.03, 0.15	0.002
24 months	3	0.5	0.18, 0.85	0.002
36 months	3	1	0.32, 1.72	0.004
42 months	3	1.3	0.39, 2.15	0.004

## Data Availability

Data generated at a central, large-scale facility are available upon reasonable request from the corresponding author.

## Supplementary Materials

The following supporting information can be downloaded at: https://www.mdpi.com/article/10.3390/cancers17060980/s1, Figure S1. Cochrane risk of bias (ROB2) evaluation: A risk of bias summary; B risk of bias graph. Figure S2. Time-varying RMSTD OS diagram for the included studies (n = 3). Figure S3. Time-varying OS hazard ratio diagram for ICG-guided (red line) versus non-ICG-guided (black line) lymphadenectomy. Figure S4. Time-varying RMSTD DFS diagram for the included studies (n = 3). Figure S5. Time-varying DFS hazard ratio diagram for ICG-guided (red line) versus non-ICG-guided (black line) lymphadenectomy. Table S1. Summary of the methodology, dose, timing, and technology for ICG-guided lymphadenectomy in the included studies. Table S2. The GRADE risk of bias tool. File S1. Search strategy. File S2. PRISMA_2020_checklist [23].

## List of Abbreviations

GC	Gastric Cancer
LN	Lymph Node
AJCC	American Joint Committee on Cancer
BMI	Body Mass Index
CI	Confidence Interval
OS	Overall Survival
DFS	Disease-Free Survival
EGJ	Esophagogastric Junction
ESMO	European Society for Medical Oncology
GRADE	Grading of Recommendations, Assessment, Development, and Evaluation
HER2	Human Epidermal Growth Factor Receptor 2
HR	Hazard Ratio
ICG	Indocyanine Green
IPD	Individual Patient Data
IPTW	Inverse Probability of Treatment Weighting
MeSH	Medical Subject Headings
MMR/MSI	Mismatch Repair/Microsatellite Instability
NCCN	National Comprehensive Cancer Network
OR	Odds Ratio
PRISMA	Preferred Reporting Items for Systematic Reviews and Meta-Analyses
RCT	Randomized Controlled Trial
RMSTD	Restricted Mean Survival Time Difference
ROB	Risk of Bias
SE	Standard Error
SMD	Standardized Mean Difference
