# Supplementary material for: Effect of Indocyanine Green-Guided Lymphadenectomy During Gastrectomy on Survival: Individual Patient Data Meta-Analysis
Source: Cancers (Basel). 2025 Mar 14;17(6):980. doi: 10.3390/cancers17060980 (PMC11940200; doi:10.3390/cancers17060980)
Supplement: Supplementary file 1 [file cancers-17-00980-s001.zip › File S1.pdf]

File S1. Search strategy

Scopus
("Gastric Cancer" OR "Gastric Carcinoma") AND ("Gastrectomy" OR "Gastric resection") AND ("ICG" OR "indocyanine" OR "fluorescence") AND ("overall survival" OR "disease free survival" OR "relapse free survival")

MEDLINE
("Gastric Cancer" OR "Gastric Carcinoma") AND ("Gastrectomy" OR "Gastric resection") AND ("ICG" OR "indocyanine" OR "fluorescence") AND ("overall survival" OR "disease free survival" OR "relapse free survival")

Web od Science
TS=("Gastric Cancer" OR "Gastric Carcinoma") AND TS=("Gastrectomy" OR "Gastric resection") AND TS=("ICG" OR "indocyanine" OR "fluorescence") AND TS=("overall survival" OR "disease free survival" OR "relapse free survival")

ClinicalTrials.gov
("Gastric Cancer" OR "Gastric Carcinoma") AND ("Gastrectomy" OR "Gastric resection") AND ("ICG" OR "indocyanine" OR "fluorescence") AND ("overall survival" OR "disease free survival" OR "relapse free survival")

Cochrane Central Library
("Gastric Cancer" OR "Gastric Carcinoma") AND ("Gastrectomy" OR "Gastric resection") AND ("ICG" OR "indocyanine" OR "fluorescence") AND ("overall survival" OR "disease free survival" OR "relapse free survival")

Google scholar
"Gastric Cancer" OR "Gastric Carcinoma" AND "Gastrectomy" OR "Gastric resection" AND "ICG" OR "indocyanine" OR "fluorescence" AND "overall survival" OR "disease free survival" OR "relapse free survival"

"Gastric Cancer" OR "Gastric Carcinoma" AND "Gastrectomy" OR "Gastric resection" AND "ICG" OR "indocyanine" OR "fluorescence" AND "overall survival" OR "disease free survival" OR "relapse free survival"
