# Supplementary material for: Effect of Indocyanine Green-Guided Lymphadenectomy During Gastrectomy on Survival: Individual Patient Data Meta-Analysis
Source: Cancers (Basel). 2025 Mar 14;17(6):980. doi: 10.3390/cancers17060980 (PMC11940200; doi:10.3390/cancers17060980)
Supplement: Supplementary file 1 [file cancers-17-00980-s001.zip › Suppl Table 1 DEF.pdf]

Author, country, year	Methodology of ICG injection	Dose	Timing of administration	Injection localization	ICG Software	ICG Brand
Wei et al., China, 2022 [31]	Endoscopic submucosal layer	ICG was diluted at a dose of 0.625 mg/ml. Subsequential injection of 0.5 ml normal saline + 0.5 mL of ICG solution + 0.5 ml normal saline	12/24h before surgery	Four quadrants around the primary tumor	PINPOINT (NOVADAQ, Stryker)	ICG (25 mg/dose, produced by Dandong Yichuang, Pharmaceutical Co., Dandong, China)
Chen et al., China, 2023 [32]	Endoscopic submucosal layer	0.5mL of the solution was injected into the submucosal layer, amounting to 2.5mg of ICG	24h before surgery	Four quadrants around the primary tumor	PINPOINT (NOVADAQ, Stryker)	1.25mg/mL ICG (Dandong Yichuang PharmaceuticalCo)
Kim et al., South Korea, 2024 [33]	Endoscopic submucosal layer	In the initial phase of the study, a total dose of 3 mg ICG was administered in four injections of 0.6 ml each at a concentration of 1.25 mg/ml from 2015 1.5 mg ICG	24h before surgery	Four quadrants around the primary tumor	PINPOINT (NOVADAQ, Stryker)	ICG; Dongindang Pharmaceutical, Incheon, South Korea

Supplementary Table S1. The table summarize the methodology, dose, timing and technology for ICG-guided lymphadenectomy in the included studies.
