# Supplementary material for: Effect of Indocyanine Green-Guided Lymphadenectomy During Gastrectomy on Survival: Individual Patient Data Meta-Analysis
Source: Cancers (Basel). 2025 Mar 14;17(6):980. doi: 10.3390/cancers17060980 (PMC11940200; doi:10.3390/cancers17060980)
Supplement: Supplementary file 1 [file cancers-17-00980-s001.zip › Suppl Table 2 DEF.pdf]

Certainty assessment							№ of patients		Effect		Certainty	Importance
№ of studies	Study design	Risk of bias	Inconsistency	Indirectness	Imprecision	Other considerations	[intervention]	[comparison]	Relative (95% CI)	Absolute (95% CI)		
Overall Survival (OS)												
3	non-randomised studies	serious	serious	not serious	not serious	all plausible residual confounding would reduce the demonstrated effect	2633	3692	-	SMD 0.54 SD higher (0.005 lower to 1.1 higher)	⨁⨁⨁◯ Moderate	CRITICAL
Disease free survival (DFS)												
3	non-randomised studies	serious	serious	not serious	not serious	all plausible residual confounding would reduce the demonstrated effect	2633	3692	-	SMD 1.26 SD higher (0.39 higher to 2.15 higher)	⨁⨁⨁◯ Moderate	CRITICAL

Supplementary Table S2. The GRADE certainty of evidence CI: confidence interval; SMD: standardised mean difference
