# Supplementary figures and images for: Effect of Indocyanine Green-Guided Lymphadenectomy During Gastrectomy on Survival: Individual Patient Data Meta-Analysis
Source: Cancers (Basel). 2025 Mar 14;17(6):980. doi: 10.3390/cancers17060980 (PMC11940200; doi:10.3390/cancers17060980)

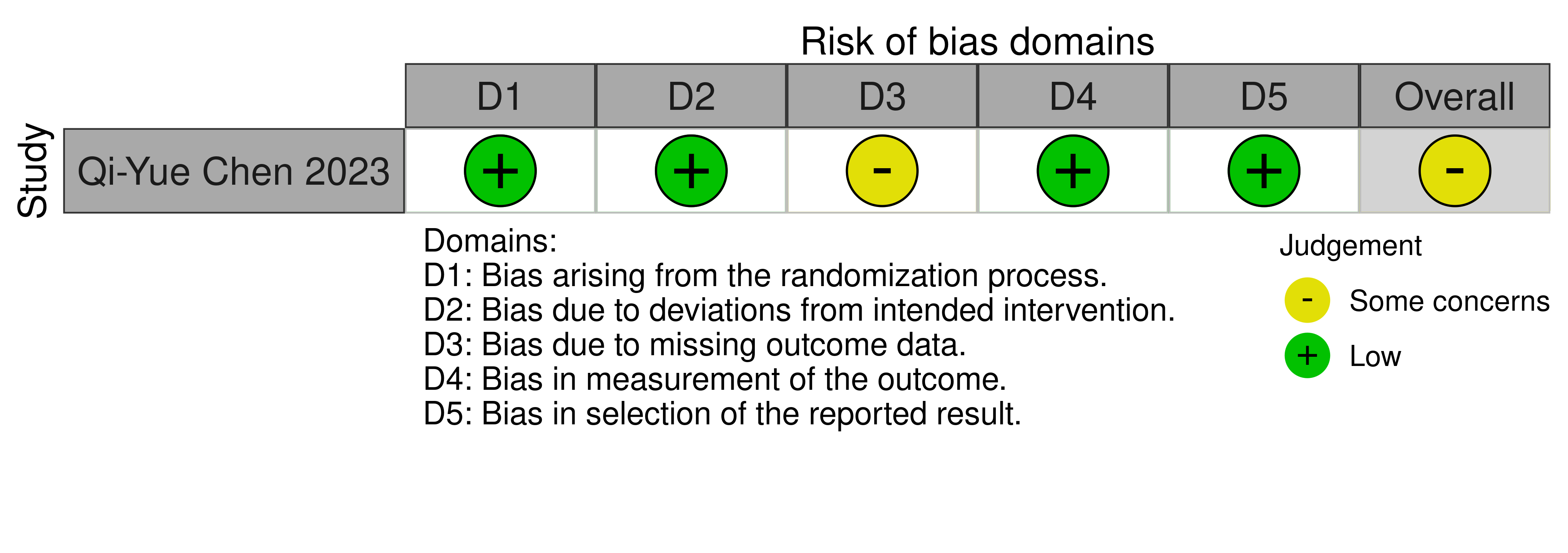

Supplement: Supplementary file 1 [file cancers-17-00980-s001.zip › Suppl Fig 1a.tiff]

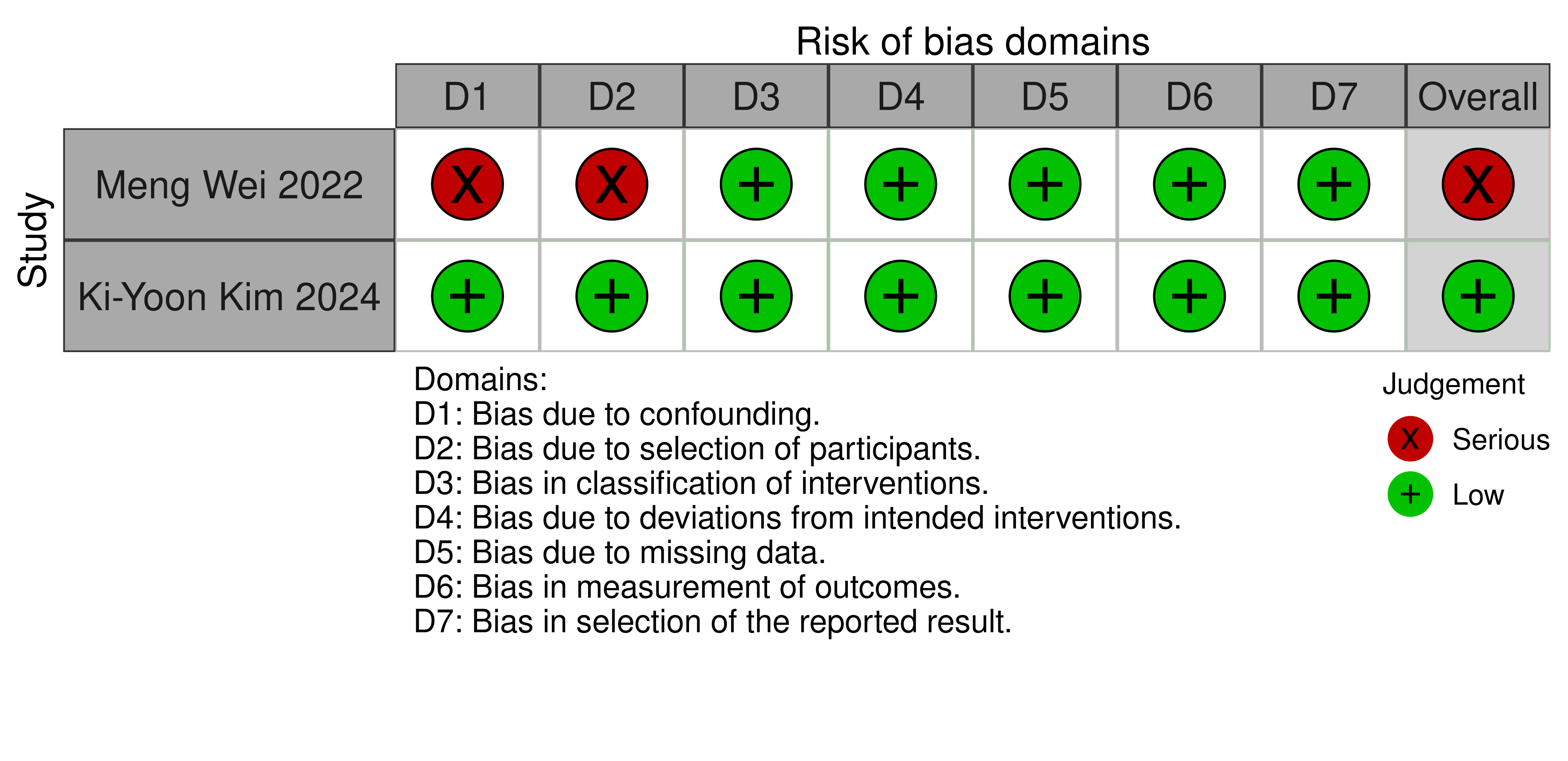

Supplement: Supplementary file 1 [file cancers-17-00980-s001.zip › Suppl Fig 1b.tiff]

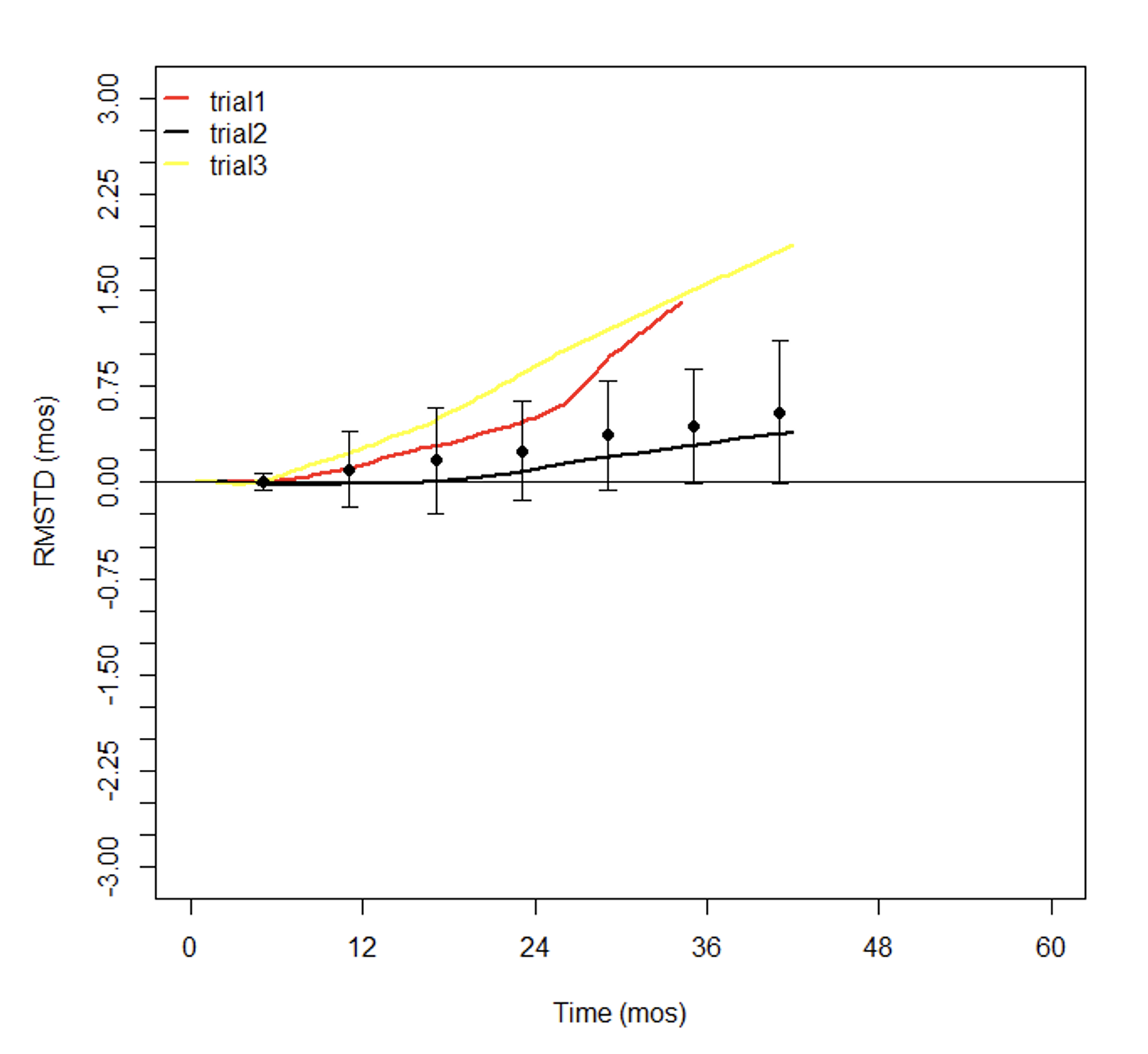

Supplement: Supplementary file 1 [file cancers-17-00980-s001.zip › Suppl Fig 2.tiff]

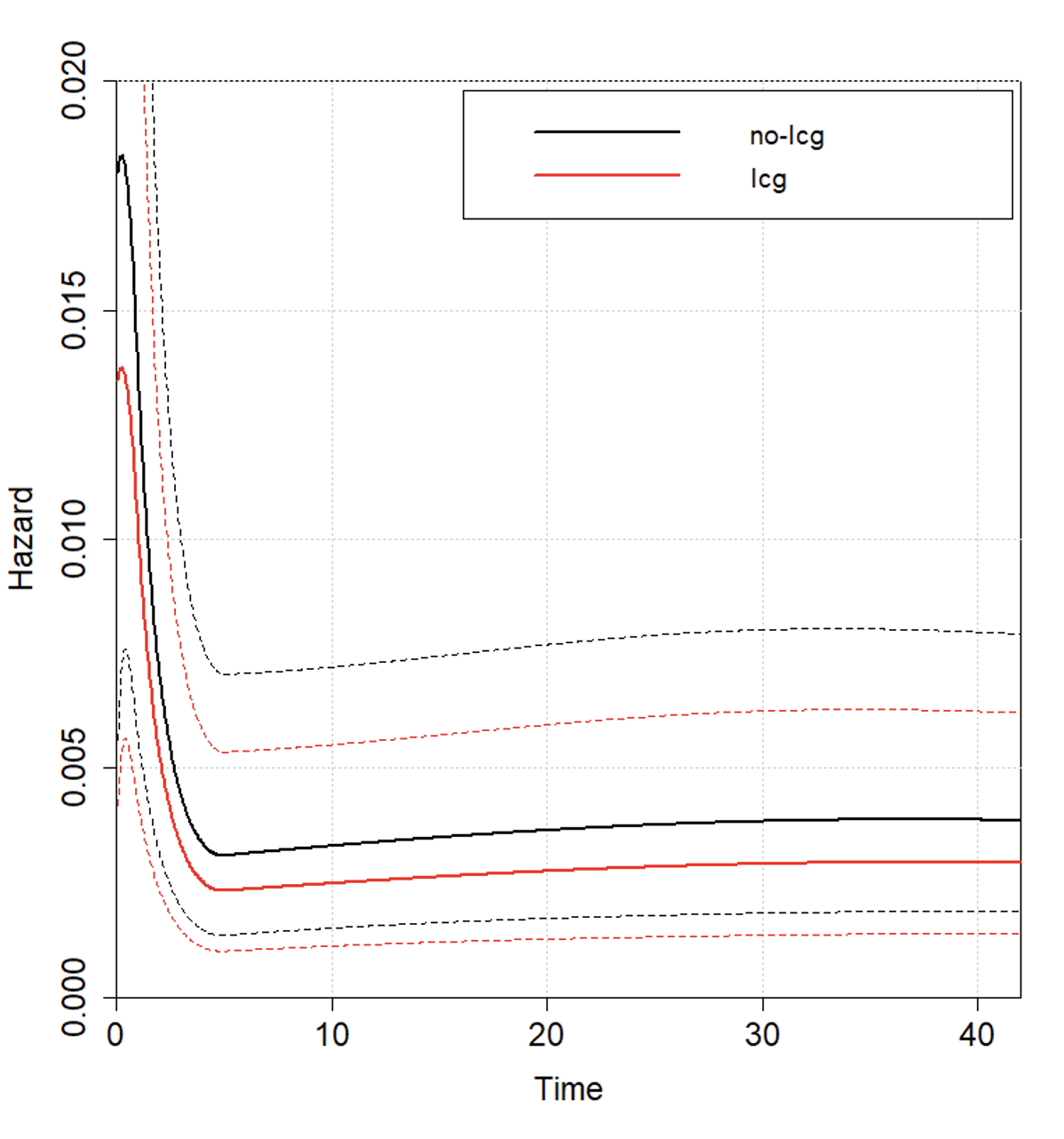

Supplement: Supplementary file 1 [file cancers-17-00980-s001.zip › Suppl Fig 3.tiff]

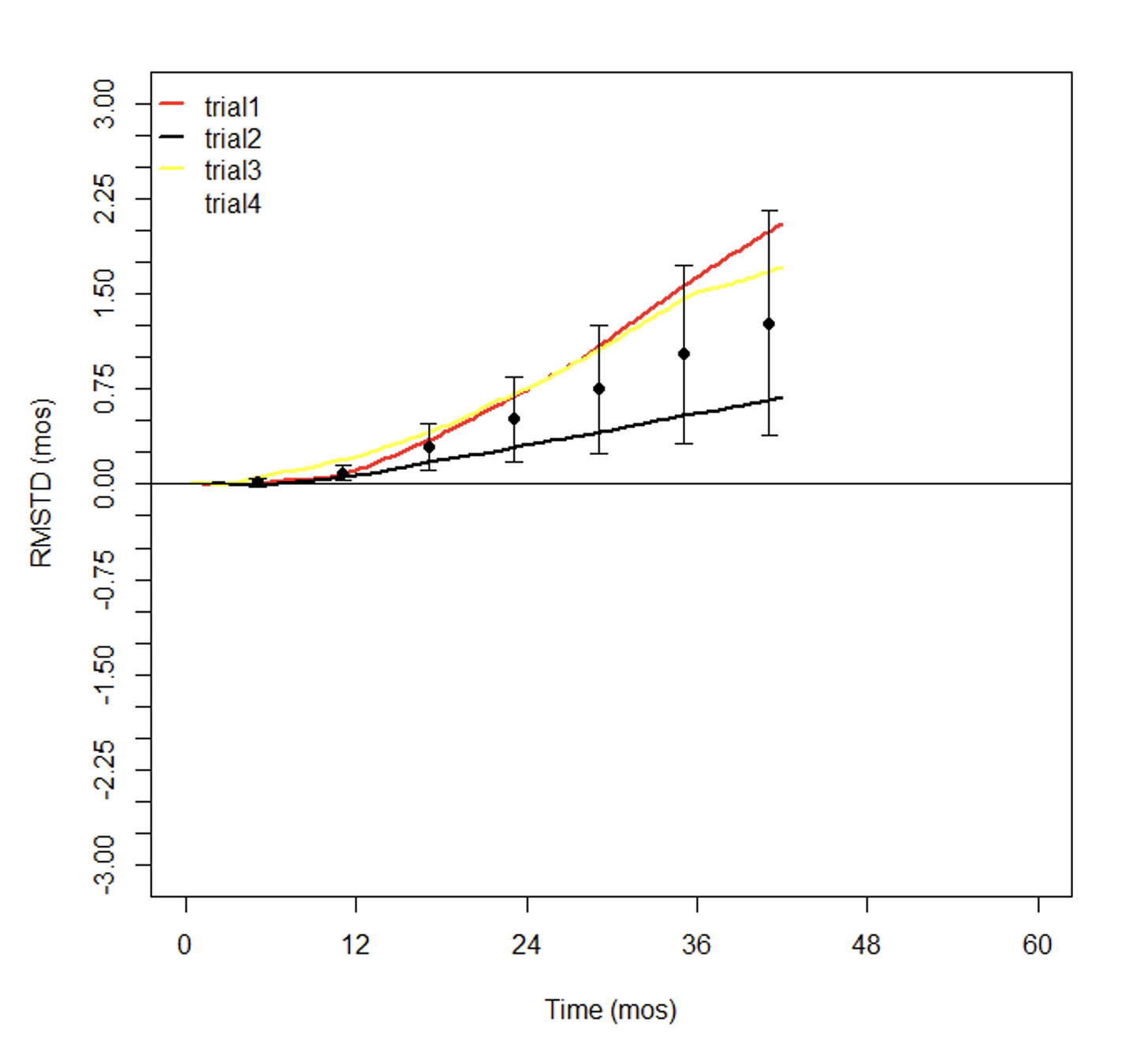

Supplement: Supplementary file 1 [file cancers-17-00980-s001.zip › Suppl Fig 4.tiff]

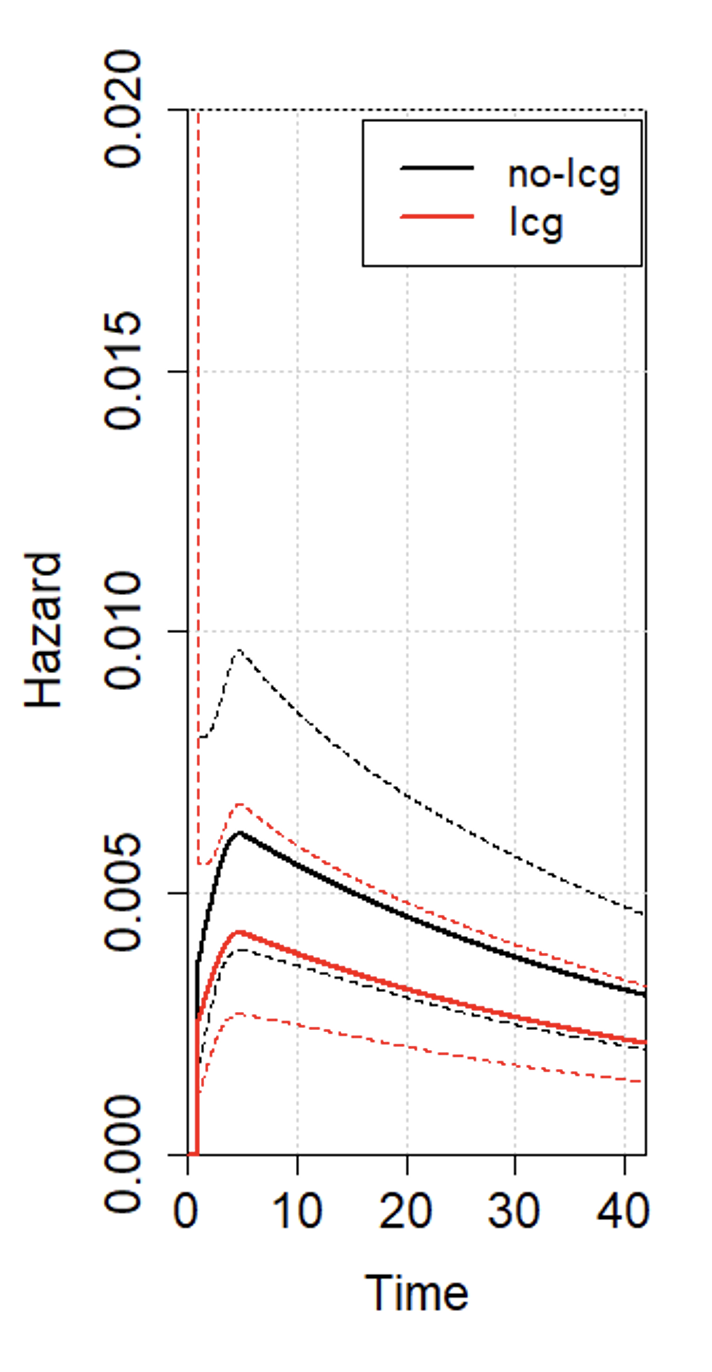

Supplement: Supplementary file 1 [file cancers-17-00980-s001.zip › Suppl Fig 5.tiff]
